# Down regulation of G protein-coupled receptor 137 expression inhibits proliferation and promotes apoptosis in leukemia cells

**DOI:** 10.1186/s12935-018-0507-1

**Published:** 2018-01-29

**Authors:** Li-Jie Men, Ji-Zhu Liu, Hai-Ying Chen, Li Zhang, Shuang-Feng Chen, Tai-Wu Xiao, Jing-Xia Wang, Guang-Yao Li, Ya-Ping Wu

**Affiliations:** 1 Department of Hematology, Liaocheng People’s Hospital and Clinical School of Taishan Medical University, Liaocheng, 252000 Shandong Province P. R. China; 2 Zhong Yuan Academy of Biological Medicine, Liaocheng University, Liaocheng People’s Hospital, Medical School of Liaocheng, Liaocheng, 252000 Shandong Province P. R. China; 3 University Medical Center Utrecht, Heidelberglaan 100, 3584 CX Utrecht, The Netherlands

**Keywords:** Leukemia, GPR137, Proliferation, Apoptosis

## Abstract

**Background:**

G protein-coupled receptors (GPR) are involved in a wide range of physiological processes, some of which, however, can be hijacked by tumor cells. Over-expression of G protein-coupled receptors 137 (GPR137) are associated with the growth of tumor cells, but under-expression of GPR137 has shown to inhibit cell proliferation in several different types of cancers. Currently, the role of GPR137 in leukemia is still unclear. In this study, the effect of under-expression of GPR137 on inhibiting the proliferation of leukemia cells is explored, to identify a novel target for leukemia treatment.

**Materials and methods:**

In this study, lentivirus-mediated RNA interference (RNAi) was employed to investigate the role of GPR137 in two leukemia cell lines K562 and HL60. The gene expression of GPR137 was analyzed by RT-PCR and its protein expression was determined by Western blot. Flow cytometry and Annexin V/7-AAD Apoptosis Detection Kit was used respectively in cell cycle and apoptosis analysis. The protein expression of CyclinD1, CDK4, BCL-2 and caspase-3 were also determined.

**Results:**

There was high level of constitutive expression of GPR137 in leukemia cancer cell lines K562 and HL60. Lentivirus-mediated RNAi could significantly down-regulate gene and protein expression of GPR137 in both cell lines. Down regulation of GPR137 was associated with the reduction in proliferation rate and colony forming capacity. In addition, down regulation of GPR137 arrested cells in the G0/G1 phase of cell cycle and induced apoptosis in both leukemia cell lines K562 and HL60.

**Conclusions:**

The expression of GPR137 is associated with the proliferation of leukemia cell lines. Down regulation of GPR137 could inhibit proliferation and promote apoptosis in leukemia cells, which makes it a promising bio-marker and therapeutic target to treat patients with leukemia.

## Background

Leukemia is the most common malignant tumor of the hematopoietic system, classified as acute myeloid leukemia (AML) and chronic myeloid leukemia (CML). In adults, the most common leukemia is AML, whose incidence increases every year, with 5-year survival rate only 50% in adults and less than 10% in patients over 70 years old [1, 2]. The abnormal epigenetics involved in the pathogenesis of AML by causing genomic instability leads to the occurrence of chromosomal abnormalities [3]. As an independent factor, age can affect the prognosis of patients, which declines significantly after the age of 60, due to a higher cytogenetic risk [4]. The incidence of CML is 0.001–0.002% [5]. Characterized by uncontrolled growth of bone marrow myeloid progenitor cells [6], CML was recognized in 1845 as a clinical condition which is associated with a specific cytogenetic change in the first class of diseases [7], and it is divided into three clinical phases: chronic phase (CP), accelerated phase (AP) and blastic crisis (BC) [8]. BCR-ABL1 signal is thought to be the driving force of CML and it may lead to changes in secondary genes. Continuous BCR-ABL1 activity induces DNA damages, resulting in clonal evolution [9], which is indicated by cytogenetic and mutational changes [10].

GPRs amplify extracellular signals to finally evoke intracellular responses. GPRs are membrane proteins on the cell’s surface. There are approximately 800 different GPR types, each of which can detect and bind specific molecules on the cell’s surface, which are called “ligands.” Upon binding a ligand, the GPR transmits a signal across the cell’s membrane where specialized, so-called G proteins work to amplify the signal using a cascade of biochemical reactions that evoke cellular responses [11]. G-protein-coupled receptors (GPRs) comprise the largest family of cell-surface molecules. They are the crucial players in multiple physiological functions by promoting cell connection through recognition of diverse ligands, like amines, bioactive peptides, nucleosides and lipids, which modulate various signaling pathways [12]. However, by proliferating independently, escaping the immune system, increasing their blood supply, invading their surrounding tissues and propagating to other organs [13], cancer cells can hijack the normal physiological functions of GPRs and participate in tumor growth and metastasis. Many GPRs-mediated reactions are not dependent on a single biochemical route, but rely on a complicated network of transduction cascades which include many physiological activities and tumor development [14].

Currently, approximately 140 GPRs are called orphan GPRs [15], whose ligands remain unidentified, therefore their function are unknown. GPR133, GPR134, GPR135, GPR136, and GPR137 are highly homologous in the prostate-specific GPR-encoding gene (PSGR) [16], whose expression in prostate tissue is restricted, but much higher in prostate tumor than in normal tissue, so it can be inferred that PSGR may act as an important gene in the development and progression of early prostate cancer [17]. GPR137 has been shown to play an important role in several different kinds of cancers [18], but its role in leukemia is currently unknown. In this study, the influence of GPR137 on the proliferation of leukemia were investigated, and whether it can be used as a therapeutic target were also explored.

## Materials and methods

### Cell culture

In this study, two human leukemia cell lines K562 and HL60 were cultured in RPMI-1640 (Hyclone, USA) supplemented with 10% FBS (Hyclone, USA), penicillin (100 U/ml and streptomycin (100 mg/ml), both of which were obtained from Institute of Hematology and Blood Diseases Hospital, Tianjin, China. Human embryonic kidney 293T cells were used to produce lentiviruses and grown in DMEM (Hyclone, USA) with 10% FBS (Hyclone, USA), penicillin (100 U/ml) and streptomycin (100 mg/ml). The cells were obtained from Central Laboratory of Liaocheng People’s Hospital, and incubated in a humidified atmosphere containing 5% CO_2_ at 37 °C.

### Lentiviral vector production and transduction

A short hairpin RNA (shRNA) targeting GPR137 was cloned into the lentiviral vector plasmid (Wuhan, China). Green fluorescent protein (GFP) reporter gene is also incorporated in the lentiviral vector to allow tracking of expressed siRNAs and to assess the transduction efficiency. Using a four-plasmid system, lentiviral vectors were produced by transient cotransfection. The lentiviral vectors expressing both GFP and shRNA (Lv-shGPR137) or control vectors expressing GFP and control shRNA (Lv-shCon) were transfected into human embryonic kidney 293T cells by lipofectamine 2000 (Invitrogen, Carlsbad, CA, USA) together with packaging plasmid pS-PAX, VSV-G and p-RSV-REV. Supernatants were collected after 48 and 72 h transfection, filtered through a 0.45 mm membrane filter and concentrated by centrifugation at 25,000 rpm for 2 h at 4 °C. The titre of lentiviruses was also determined.

In transduction experiments, 5 × 10^5^ K562 and HL60 cells were seeded in 6-well plates, and then transduced with lentivirus in the presence of 6 mg/ml of polybrene for 6 h. After 24 h, the medium was changed.

### Real Time-PCR analysis

Real Time-PCR analysis was used to evaluate the gene expression of GPR137. K562 and HL60 cells were transduced with lentivirus for 5 days, then total RNAs were extracted using the Trizol reagent (Invitrogen, Carlsbad, CA, USA). According to the manufacturer’s instructions, cDNA was reverse transcribed from isolated RNA using a PrimeScript RT reagent kit (Takara, China). GPR137 mRNA expression was evaluated by PCR using SuperReal PreMix Plus (SYBR Green, Takara, China) on ABI 7500 system (Applied Biosystems, Foster City, CA, USA). The PCR reaction system was performed in a final volume of 20 μl containing 2 μl cDNA, 0.4 μl forward and reverse primers (10 μM),10 μl SYBR premix exTaq (Takara, China), ROX Reference Dye II 0.4 μl and ddH_2_O 6.8 μl. The PCR conditions were: 95 °C for 3 min, 40 cycles of 95 °C for 12 s and 65 °C for 40 s. The primer sequences of GPR137 gene used in RT-PCR and housekeeping gene GAPDH were as follows:

- GPR137 forward, 5′-TTGACGCTTATGAA CTCTACTTT-3′
- and reverse 5′-CGCAGATGACGAACAGGGA-3′;
- GAPDH forward, 5′-GGACACTGAGCAAGAGAGGC-3′
- and reverse 5′-TTATGGGGGTCTGGGATGGA-3′.

GAPDH was used as an internal control. Relative gene expression levels were determined and presented as fold changes. The experiment was independently repeated for three times.

### Western blot

Western blotting analysis was used to determine relative protein expression. After K562 cells and HL60 were transduced with the lentivirus for 5 days, the cells were washed twice with ice-cold phosphate buffered saline (PBS) and were harvested using radio immune precipitation assay kit (RIPA) with inhibitor phenyl methane sulfonyl fluoride (PMSF). After lysis for 30 min, the cellular extracts were centrifuged at 12,000*g* for 15 min at 4 °C. The protein concentrations were quantified by the BCA assay kit (Beyotime Biotechnology, Jiangsu, China). Equal concentrations of each protein sample (20 μg) was boiled for 5 min in the loading buffer and loaded onto a 10% sodium dodecyl sulphate-polyacrylamide gel electrophoresis SDS-PAGE. Then the proteins were transferred onto a polyvinylidene difluoride (PVDF) membranes (Millipore, USA) at 40 V for 50 min. After that, the membranes were blocked in Tris Buffered Saline Tween (TBST) containing 5% non-fat milk and 0.1% Tween for 70 min. Rabbit anti-GPR137 poly-clonal antibodies (1:1000; Abcam, USA), Rabbit anti-CyclinD1 poly-clonal antibodies (1:1000; CST, USA), rabbit anti-CDK-4 poly-clonal antibodies (1:1000; CST,USA), rabbit anti-BCL-2 poly-clonal antibodies (1:1000; Abcam, USA), rabbit anti-caspase 3 poly-clonal antibodies (1:1000; Abcam, USA), mouse anti-β-actin, (1:2000; Beyotime Biotechnology, Jiangsu, China) were incubated for 12 h at 4 °C. Following overnight incubation with the primary antibodies, membranes were washed three times with TBST for 10 min. The membrane was incubated with the goat anti-rabbit secondary antibody (Beyotime Biotechnology, Jiangsu, China) at 1:4000 for 40 min at room temperature. The target protein was finally visualized using an enhanced chemiluminescence (ECL) system (Beyotime Biotechnology, Jiangsu, China). Each experiment was repeated three times and anti-β-actin antibody was used as loading controls. The results of western blot were analyzed by Image-Pro Plus software 6.0 (Bio-Rad, USA).

### Cell viability assay

Cell viability was assessed using CCK-8 assay kit (Dojindo Laboratories, Kumamoto, Japan). Five days after lentivirus transduction, 2 × 10^3^ transduced K562 and HL-60 cells were seeded into 96-well plates and cultured in RPMI1640 medium containing 10% FBS at 37 °C in 5% CO_2_ atmosphere for 1, 2, 3, 4 and 5 days, respectively. Briefly, 10 μl of CCK-8 solution was added to each well and incubated for 2 h. The cell viability in each well was measured at an absorbance of 450 nm using a spectrophotometer according the manufacturer’s instruction. All experiments were performed in triplicate.

### Colony formation assay

Lv-shGPR137 K562 and HL60 cells were seeded into a 6-well plate with 500 cells/well and cultured in RPMI 1640 with 10% heat inactivated fetal bovine serum (FBS, Hyclone, USA) and 0.9% methylcellulose (Sigma, USA) in a humidified atmosphere containing 5% CO_2_ at 37 °C for 10 days. Cells were washed twice with PBS and fixed with 4% paraformaldehyde for 30 min at room temperature. The colonies were then stained with freshly prepared diluted Giemsa for 10 min. After being washed and air-dried for three times, the total number of colonies (> 50 cells/colony) were counted under the microscope.

### Cycle progression analysis

Being transduced with lentivirus for 5 days, K562 and HL60 cells were collected by centrifugation at 1000 rpm for 5 min and then counted. The cells were then washed with cold phosphate buffered saline (PBS) and suspended in 950 µl of cold 70% ethanol. Next, the cells were washed with cold PBS and suspended in 950 µl of cold 70% ethanol. After being incubated at 4 °C for 30 min, cells were collected by centrifugation and resuspended in iodide buffer and incubated at 37 °C for 30 min in dark. Finally, the stained cells were analyzed by Coulter flow cytometry (Becton–Dickinson, San Jose, CA). Each experiment was repeated three times.

### Apoptosis analysis

The apoptosis of the cells was measured using Annexin V-APC/7-AAD double staining (BD Pharmingen, USA) by flow cytometry. After transduced with lentivirus for 5 days, the cells were collected and then washed twice with cold PBS. Later, the cells were re-suspended in binding buffer at a concentration of 1 × 10^6^ cells/ml. Each 100 μl of the solution (1 × 10^5^ cells) was transferred to a 5 ml culture tube. Five microlitre of APC Annexin V and 5 μl of 7-AAD were added. Then cells were incubated for 15 min at 25 °C in the dark and analyzed by flow cytometry within 1 h Each experiment was repeated three times.

### Statistical analysis

Data from experiments were collected from at least three independent experiments and then presented as mean ± SD. Statistical analysis was calculated by *t* test or one way analysis of variance. P < 0.05 was considered to indicate a significant difference. The statistical analysis was performed using the SPSS 17.0 statistical software package.

## Results

### Lentiviral transduction of leukemia cell lines

**Fig. 1:**
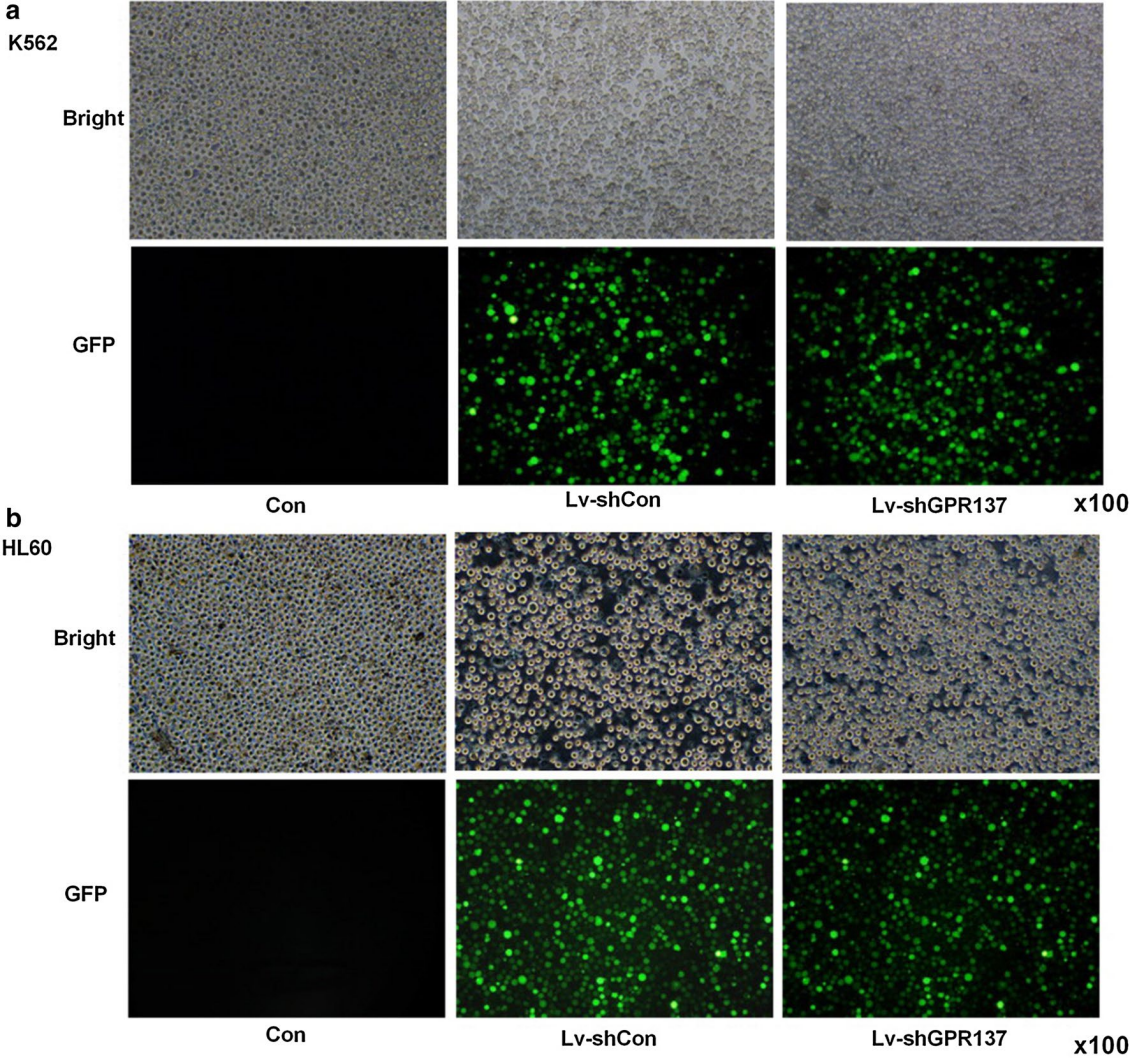
Representative images of K562 (**a**) and HL60 (**b**) cells 5 days of after lentivirus transduction. Cells were either nontransduced (Con) or transduced with control lentiviral vectors (Lv-Con) or lentiviral vectors expressing shGPR137 (Lv-shGPR137) (magnification, ×100). The transduction efficiency was greater than 90%

Lentiviral vectors could transduce both cell lines efficiently. The transduction efficiency for lentiviral vectors expressing GPR137 (Lv-shGPR137) or control vectors which expressing GFP and control shRNA (Lv-shCon), was greater than 90% (Fig. 1).

GPR137 mRNA and its protein expression were inhibited by lentivirus-mediated shRNA expression in K562 and HL60 cells.

**Fig. 2:**
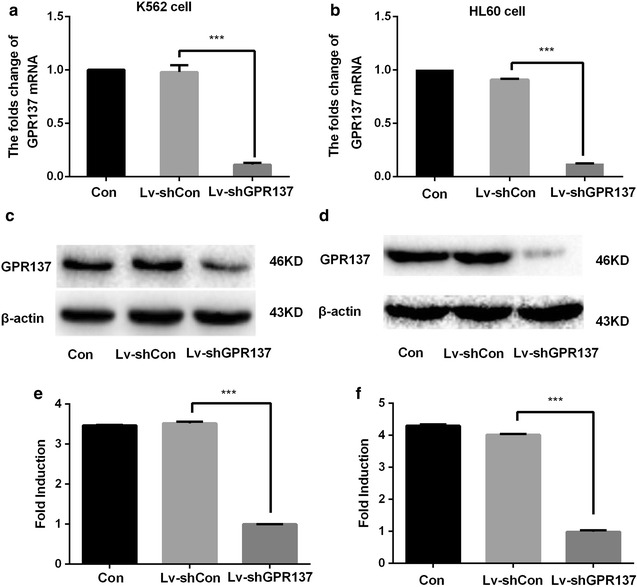
RT-PCR analysis of mRNA levels of GPR137 in K562 (**a**) and HL60 (**b**) cells. The cells were either nontransduced (Con), or transduced with control lentiviral vector (Lv-shCon) or lentiviral vector expressing shGPR137 (Lv-shGPR137). Representative Western blot and quantification of GPR137 protein levels in K562 (**c**) and HL60 (**d**) cells. **e**, **f** the relative protein expressions of **c** and **d**. The cells were whether nontransduced, or transduced with Lv-shCon or Lv-shGPR137. The protein expression was normalised by β-actin expression. There was a significant difference between Con and Lv-shGPR137 (P < 0.001). *P < 0.05, **P < 0.01, ***P < 0.001, compared with Con or Lv-shCon cell line

As demonstrated by western blot analysis (Fig. 2c, d), GPR137 was constitutively expressed in both cell lines, which indicates that it may be associated with tumorgenesis. Using lentiviral vector delivery, RNAi could suppress the expression of GPR137 in both cell lines. Compared with the Control or Lv-shCon group, the expression of GPR137 mRNA has decreased up to 90% in cells transduced with Lv-shGPR137 (P < 0.001, Fig. 2a, b). There was no significant difference between Control and Lv-shCon group.

Accordingly, the level of GPR137 protein expression was also significantly reduced in Lv-shGPR137 group (Fig. 2c, d). The protein expression of GPR137 in Lv-shGPR137 group and Lv-shCon group was 23.14 ± 0.04% and 81.60 ± 0.55%, respectively (P < 0.0001) in K562 cells. The protein expression of GPR137 was 17.30 ± 0.48% in HL60 transduced with Lv-shGPR137 and was 80.38 ± 0.29% in Lv-shCon group (P < 0.001). There was no significant difference between Control and Lv-shCon group.

### Down regulation of GPR137 inhibits cell proliferation in K562 and HL60 cells

**Fig. 3:**
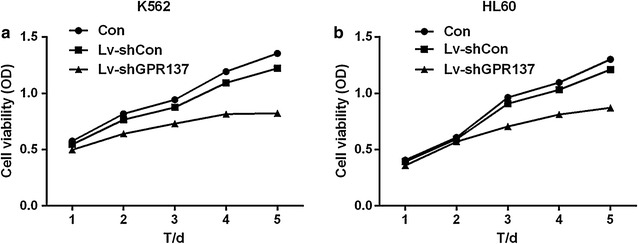
Down regulation of GPR137 inhibits K562 (**a**) and HL60 (**b**) cells proliferation. The cell viability was assessed by CCK-8 assay. The cells were either nontransduced (Con), or transduced with control lentiviral vector (Lv-shCon) or lentiviral vector expressing shGPR137 (Lv-shGPR137). The proliferation rates of cells transduced with Lv-shGPR137 were significantly decreased compared to nontransduced cells or cells transduced with Lv-shCon

To elucidate the role of GPR137 knockdown in K562 and HL60 cell proliferation, CCK-8 assay was conducted in K562 and HL60 cells which were transduced with Lv-shCon and Lv-shGPR137 5 days in advance. Compared with Control and Lv-shCon group, cell viability was significantly inhibited in both K562 and HL60 cells in Lv-shGPR137 group (Fig. 3a, b), suggesting that GPR137 is associated with the proliferation of K562 and HL60 cells. The results were also indicated in the form of proliferative index (Table 1).

**Table 1:** The proliferative index of K562 and HL60 cells

Time/day	Proliferative index (PI)			
	K562 cells		HL60 cells	
	Lv-shCon	Lv-shGPR137	Lv-shCon	Lv-shGPR137
1	0.95	0.87**	0.97	0.89*
2	0.94	0.78**	0.97	0.94*
3	0.93	0.77***	0.94	0.73***
4	0.92	0.68***	0.94	0.76***
5	0.90	0.61**	0.93	0.67***

* P < 0.05, ** P < 0.01, *** P < 0.001

### Down regulation of GPR137 reduces colony formation ability in K562 and HL60 cells

**Fig. 4:**
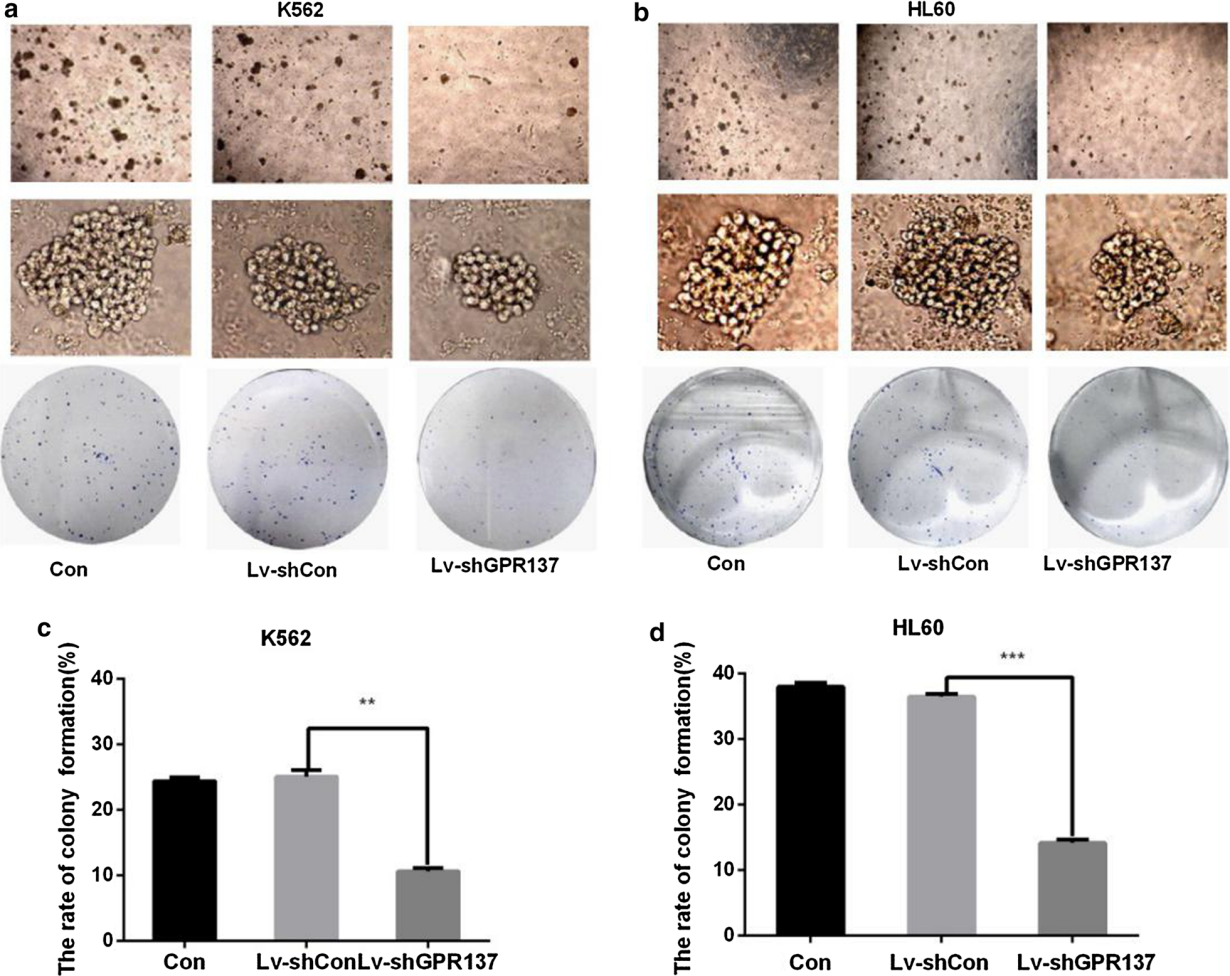
Down regulation of GPR137 inhibits the colony formation capacity of K562 and HL60 cells. Representative images of the size and numbers of colonies in K562 (**a**) and HL60 (**b**) cells either nontransduced, or transduced with control lentiviral vector (Lv-shCon) or lentiviral vector expressing shGPR137 (Lv-shGPR137). The rate of clone formation in K562 (**c**) and HL60 (**d**) cells with Giemsa staining are also presented. The data was represent as mean ± SD of (three) experiments. *P < 0.05, **P < 0.01, ***P < 0.001, compared to cells transduced with Lv-shCon

Compared with Control and Lv-shCon group, the rate of clone formation and the size of single colony were significantly reduced in K562 and HL60 cells in Lv-shGPR137 group (Fig. 4a–d), which indicates that knockdown of GPR137 could suppress the colony formation of K562 and HL60 cells.

### Down regulation of GPR137 arrests cell cycle progression in K562 and HL60 cells

**Fig. 5:**
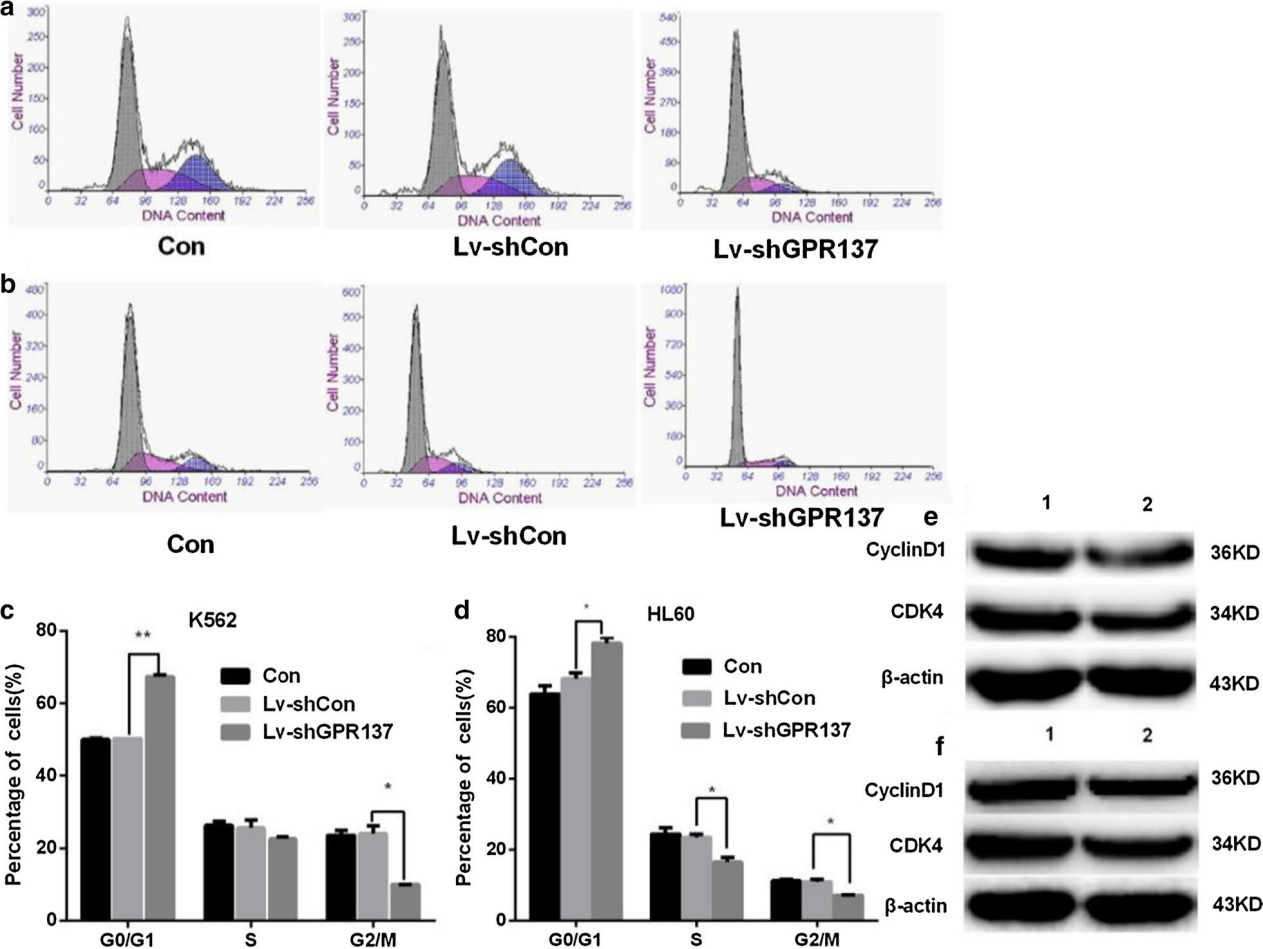
Representative flow cytometric histograms of distribution of cell cycles of K562 (**a**) and HL60 (**b**). down regulation of GPR137 leads the cell-cycle arrested at G0/G1 phase of cell cycle in both cell lines. Percentages of K562 (**c**) and HL60 (**d**) cells at G0/G1, S, G2/M phase were shown. There was a significant difference between cells transduced with control lentiviral vector (Lv-shCon) and cells transduced with lentiviral vector expressing GPR137 (LvshGPR137) (P < 0.05). The expression of CyclinD1 and CDK4 were reduced in K562 (**e**) and HL60 (**f**) cells. Lane 1: nontransduced cells and lane 2: cells transduced with lentiviral vector expressing shGPR

Cells was first labeled with propidium iodide (PI), and then the distribution of K562 (Fig. 5a) and HL60 (Fig. 5b) cells from different phases of was analyzed using flow cytometry. As shown, the percentages of G0/G1 in K562 (Fig. 5c) and HL60 (Fig. 5d) were significantly higher when cells transduced with Lv-shGPR137 than that in non-transduced cells or cells transduced with control vector Lv-shCon. For both cell lines, the percentage of cells in G2 and S phases was dramatically decreased in cells transduced with Lv-shGPR137 than that in non-transduced cells and cells transduced with Lv-shCon (P < 0.05). No significant difference was observed between non-transduced cells and cells transduced with Lv-shCon. The expression of CyclinD1 and CDK4 were also reduced in K562 (Fig. 5e) and HL60 (Fig. 5f) cells transduced with Lv-shGPR137. These results indicated that the decreased cell proliferation and colony formation capacity was associated with knockdown of GPR137 induced cell cycle arrest at G0/G1 phase.

### Down regulation of GPR137 promotes cell apoptosis in K562 and HL60 cells

**Fig. 6:**
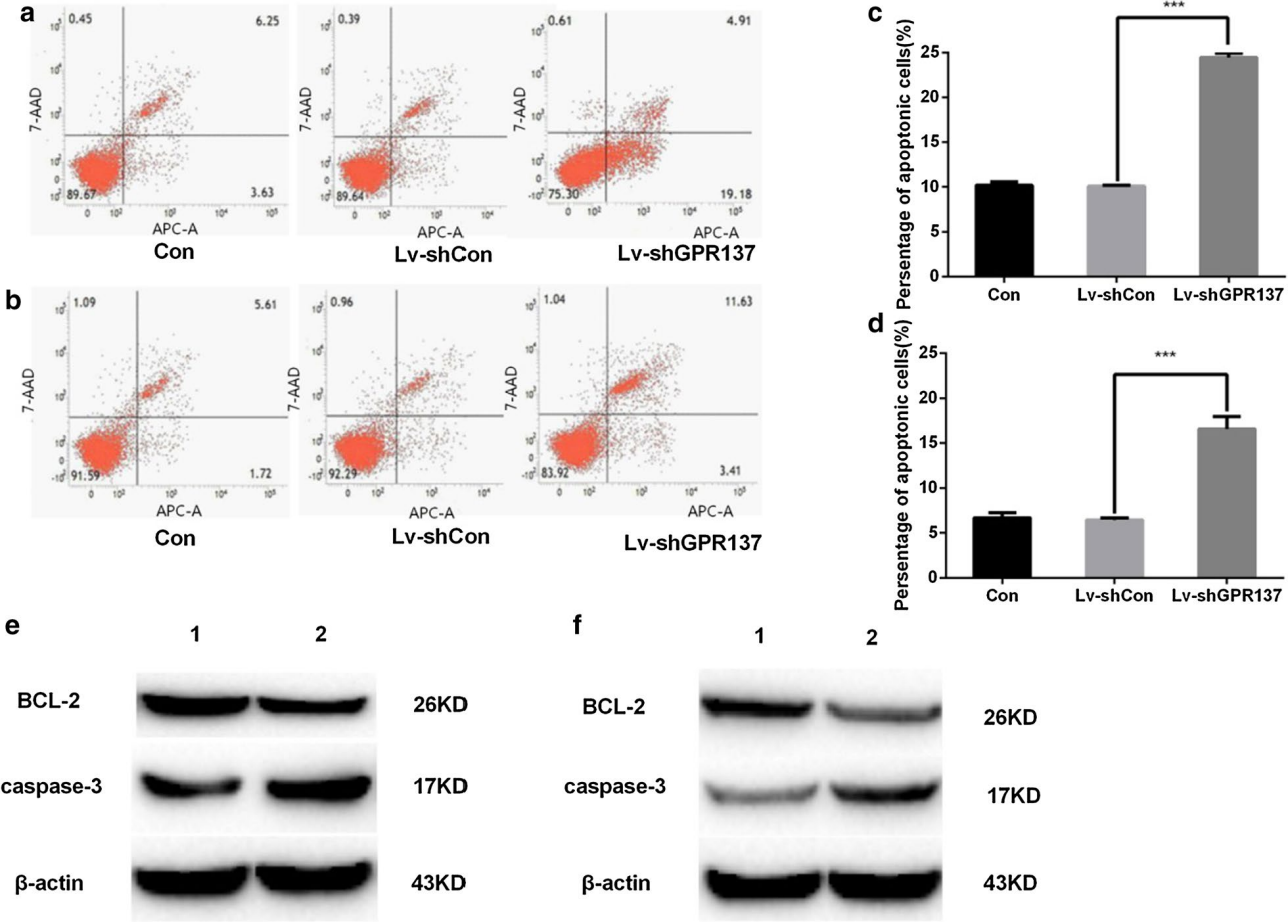
Representative of flow cytometric analysis of apoptosis with Annexin V and 7-AAD (**a**, **b**). The percentages of apoptotic cells were quantified (**c**, **d**). down regulation of GPR137 promote apoptosis in K562 (**a**, **c**) and HL60 (B, D) cells. There was a significant difference between cells transduced with control lentiviral vectors and those with lentiviral vectors expressing GPR137 (P < 0.001). Representative Western blot of BCL-1 and Caspase 3 expression in K562 (**e**) and HL60 (**f**). Lane 1 was cells transduced with control lentiviral vectors and lane 2 was cells transduced with lentiviral vectors expressing GPR137. The expression of BCL-2 was reduced and the expression of Caspase-3 increased in K562 (**e**) and HL60 (**f**) cellsCon: cells without infection; Lv-shCon: cells infected with the Lv-shCon; Lv-shGPR137: cells infected with the LvshGPR137; 1: Lv-shCon, 2: Lv-shGPR137. *P < 0.05, **P < 0.01, ***P < 0.001, compared with Con or Lv-shCon cell line

Many physiological and pathological processes involve a balance between apoptosis and cell proliferation. To examine whether knockdown of GPR137 influences apoptosis in K562 and HL60 cells, the Annexin V/7-AAD double staining was performed in both cell lines using a flow cytometer (Fig. 6a, b). The data revealed that a significantly increased apoptosis was observed in both cell lines transduced with Lv-shGPR137, compared with that of non-transduced cells or cells transduced with Lv-shCon (Fig. 6c, d). Consistent with this, the expression of BCL-2 reduced but the expression of Caspase-3 increased in both K562 (Fig. 6e) and HL60 (Fig. 6f) cells when transduced with Lv-shGPR137. Therefore, these results suggest that down regulation of GPR137 may promote apoptosis in K562 and HL60 cells.

## Discussion

Leukemia is characterized by over production of abnormal blast cells in the bone marrow that often spill out into other hematopoietic tissues. However, the development of cancer is a very complicated process, involving a variety of genetic and epigenetic changes [19]. Despite the diversity and heterogeneity of tumors, they all share the characteristics of unrestricted proliferation. Recent advances in molecular target therapy offer a new strategy to target a specific gene over-expressed in tumor cells and therefore inhibit tumor proliferation [20]. So far, several therapeutic agents were used to interfere with the expression of the target gene, including ASO (anti-sense oligonucleotides), ribozyme and siRNA, which demonstrated promising therapeutic effects on tumor formation, growth and metastasis [21].

GPRs have been implicated as a critical player in cancer biology, which can be divided into two groups: the odorant and non-odorant. Odorant GPRs are limited in the specialized cells to detect external cues, tastes, smells and pheromones, and regulate the behaviour of the organism, such as feeding and mating. Expressed throughout the organism, non-odorant GPRs are binding on diverse endogenous ligands and modulate the host of various physiological processes, including hematopoiesis, hemostasis, neurotransmission, cardiac function, vascular tone, metabolism and reproduction immune function [22]. As key signal transduction conduits, GPRs link extra-cellular inputs with diverse cellular responses [23]. Over-expression of GPR is associated with tumorigenicity, and several GPRs such as GPR30, GPR87 and GPR110, are implicated in tumor growth and metastasis [24]. GPR30 was over-expressed in solid tumors including breast, ovarian and endometrial cancer [25]. GPR87 was over-expressed in diverse cancers including lung, cervix, skin, urinary bladder, testis, and head and neck cancers [26]. GPR110 was over-expressed in lung and prostate cancer [27]. Therefore, targeting GPRs genes may provide a promising approach for the diagnosis and treatment of tumor.

GPR137 has been reported to be involved with the proliferation of tumor cells in several cancers, including gastric, colon, pancreatic, hepatoma, urinary bladder cancer, medulloblastoma and malignant glioma [28]. However, the functions of GPR137 in leukemia were previously unknown. It is shown in this study that there was high level of constitutive expression of GPR137 in leukemia cells. Using siRNA technology, GPR137 was demonstrated to be associated with the proliferation of leukemia cells.

Through lentivirus-mediated silenced system, the silence of GPR137 gene and its protein expression could significantly inhibit cell proliferation and colony-formation capacity. The decreased proliferation was associated with silenced GPR137-induced cell cycle arrest, which causes leukemia cells arrest in G0/G1 phase of cell cycle. Cell cycle control disorder is the main reason to tumorigenesis. As one of the most important proteins to regulate cell cycle, Cyclin D1 can activate CDK4, which allows the cell cycle to progress through G1 into S [29], thus promoting the development of various cancers. It was shown that both Cylin D1 and CDK4 were reduced in leukemia cells when GPR137 expression was silenced. The cell apoptosis is a regulated cellular suicide program critical for metazoan development [30]. Caspase-3 mediates the most critical process of apoptosis execution molecule [31]. When GPR137 was silenced, Caspase-3 increased, suggesting that GPR may play a role in the interconnection of cell proliferation and apoptosis.

## Conclusions

It is shown that GPR137 was constitutively expressed in leukemia cells, and through leading cells in G0/G1 phase, its silence inhibited cell proliferation and promoted apoptosis in K562 and HL60 cells, which indicates that targeting GPR137 may serve as a potentially novel approach for the diagnosis and therapy of leukemia.

## Abbreviations

GPR: G protein-coupled receptor
GPR137: G protein-coupled receptor 137
RNAi: RNA interference
AML: acute myeloid leukemia
CML: chronic myeloid leukemia
GFP: green fluorescent protein
AP: accelerated phase
BC: blastic crisis
PSGR: prostate-specific G-protein-coupled receptor
shRNA: short hairpin RNA
RIPA: radio immune precipitation assay kit
PMSF: phenyl methane sulfonyl fluoride
PVDF: polyvinylidenedifluoride
TBST: tris buffered saline tween
ECL: enhanced chemi luminescence
PBS: phosphate buffered saline
PI: propidium iodide
